# Encapsulation of gold nanoparticles into self-assembling protein nanoparticles

**DOI:** 10.1186/1477-3155-10-42

**Published:** 2012-10-31

**Authors:** Yongkun Yang, Peter Burkhard

**Affiliations:** 1 Institute of Materials Science, University of Connecticut, 97 N. Eagleville Road, Storrs, Mansfield, CT, 06269, USA; 2 Department of Molecular and Cell Biology, University of Connecticut, 91 N. Eagleville Road, Storrs, Mansfield, CT, 06269, USA

## Abstract

**Background:**

Gold nanoparticles are useful tools for biological applications due to their attractive physical and chemical properties. Their applications can be further expanded when they are functionalized with biological molecules. The biological molecules not only provide the interfaces for interactions between nanoparticles and biological environment, but also contribute their biological functions to the nanoparticles. Therefore, we used self-assembling protein nanoparticles (SAPNs) to encapsulate gold nanoparticles. The protein nanoparticles are formed upon self-assembly of a protein chain that is composed of a pentameric coiled-coil domain at the N-terminus and trimeric coiled-coil domain at the C-terminus. The self-assembling protein nanoparticles form a central cavity of about 10 nm in size, which is ideal for the encapsulation of gold nanoparticles with similar sizes.

**Results:**

We have used SAPNs to encapsulate several commercially available gold nanoparticles. The hydrodynamic size and the surface coating of gold nanoparticles are two important factors influencing successful encapsulation by the SAPNs. Gold nanoparticles with a hydrodynamic size of less than 15 nm can successfully be encapsulated. Gold nanoparticles with citrate coating appear to have stronger interactions with the proteins, which can interfere with the formation of regular protein nanoparticles. Upon encapsulation gold nanoparticles with polymer coating interfere less strongly with the ability of the SAPNs to assemble into nanoparticles. Although the central cavity of the SAPNs carries an overall charge, the electrostatic interaction appears to be less critical for the efficient encapsulation of gold nanoparticles into the protein nanoparticles.

**Conclusions:**

The SAPNs can be used to encapsulate gold nanoparticles. The SAPNs can be further functionalized by engineering functional peptides or proteins to either their N- or C-termini. Therefore encapsulation of gold nanoparticles into SAPNs can provide a useful platform to generate a multifunctional biodevices.

## Background

Due to their unique size-dependent properties, inorganic nanoparticles and their applications in the life sciences have been a topic of dramatically increasing interest over the last several years [1,2]. Gold nanoparticles (GNPs) are the most commonly used inorganic nanoparticles for biological applications [2,3], because of their attractive physical and chemical properties [4]. GNPs have been mainly used for labeling and visualizing applications as they can strongly absorb and scatter visible light. This is because of their surface plasmon resonance [5]. GNPs are often used as contrast agents for transmission electron microscopy and X-ray imaging because of their ability to scatter electrons and X-rays efficiently [6]. GNPs generate heat when they absorb light, which enables their potential in photo-thermal therapeutic applications [7,8]. GNPs are also promising as drug and gene delivery vehicles [9]. For example, they have been used as nano-bullets for gene guns [10]. In addition, GNPs are inert and relatively biocompatible [11]. They can easily be synthesized and conjugated with biological molecules in a straightforward manner [4].

The uses of GNPs in biological applications have demonstrated the importance of the conjugation of GNPs with biological molecules [12,13]. The biological molecules not only provide the interfaces for interactions between nanoparticles and biological environment, but also contribute their biological functions, such as tumor cell targeting [14], cell penetration [15], antibody-antigen recognition [16], and many others. Furthermore, biological molecules, such as DNA, can serve as platform for assembly and organization of GNPs [1,17]. Due to their well-defined surface chemistry [4], GNPs can be modified and functionalized with a wide variety of biological molecules, such as peptides [18], proteins [19], oligonucleotides [1,17], carbohydrates [20], and even whole viral capsids [21-26]. Although several publications reported that GNPs with a defined number of attached molecules per particle could be obtained using sorting techniques [27,28], no protocols for controlling the exact number of attached molecules per gold particles have yet been established [29,30]. The viral capsids provide an ordered and controlled platform for conjugation with GNPs [31,32]. However, the relatively stringent structure of the viral capsids limits their further functionalization via fusion with functional peptides [33].

**Figure 1:**
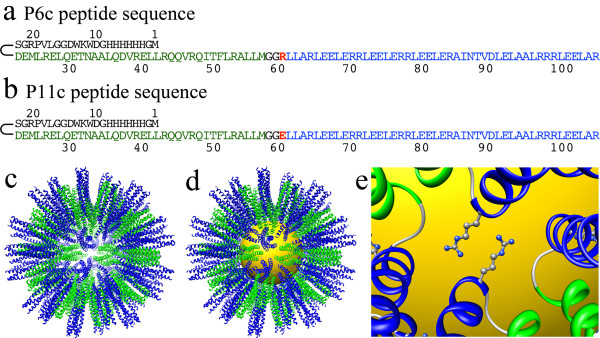
**Computer models for the encapsulation of GNPs into SAPNs.** (**a**) The sequence of P6c protein. (**b**) The sequence of P11c protein. (**c**) Computer model of the T = 3 icosahedral SAPN formed by self-assembling 180 P6c protein chains. The central cavity has a size of approximately 10 nm. The thickness of the protein shell is about 9 nm. Blue: trimeric coiled-coil domain. Green: pentameric coiled-coil domain. The his-tag is not shown. (**d**) Computer model of the encapsulation of gold nanoparticle by the P6c SAPN. Blue: trimeric coiled-coil domain. Green: pentameric coiled-coil domain. The his-tag is not shown. The size of the gold nanoparticle is around 10 nm (golden-colored ball in the center). (**e**) The zoom-in model of the side chains from arginine residues (R61 in P6c). These side chains results in a highly positively charged central cavity, and they might have interactions with GNPs.

In this paper, we present the use of self-assembling protein nanoparticles (SAPNs) [34,35] to encapsulate GNPs. The design principle of the SAPNs was inspired from the symmetrical structure of viral capsids. To mimic icosahedral viral capsids, Raman et al. developed a strategy to construct a nanoparticle based on the self-assembling properties of coiled-coil oligomerization domains [35]. We recombinantly expressed the P6c protein (Figure 1a), which has an N-terminal 36-amino-acid pentameric domain from the slightly modified cartilage oligomerization matrix protein linked by two glycine residues to a 46-amino-acid de novo designed trimeric domain. In our previous works [34], we found that the majority of the SAPNs formed by the P6c proteins have T = 3-like icosahedral structure, although the SAPNs are composed of multiple species with different numbers of co-assembled proteins. The T = 3-like icosahedral model shows that 180 protein chains self-assemble into SAPNs with a shell of about 9 nm and a central cavity of about 10 nm (Figure 1c). The environment in the central cavity can easily be modified by site directed mutagenesis. For instance, the SAPNs can have a positively charged (P6c in Figure 1a) or negatively charged (P11c in Figure 1b) central cavity. The central cavity is ideal to encapsulate GNPs with similar size. Here we describe the ability of SAPNs to encapsulate commercially available GNPs that have different sizes and surface coatings. The SAPNs have a regular structure and can easily be functionalized by fusing functional peptides to the termini of the P6c protein. Therefore, the SAPNs provide a promising tool for the functionalization of GNPs. 

## Results

### Encapsulation of citrate-coated GNPs by P6c SAPNs

GNPs of the three different sizes of 5, 10 and 15 nm with citrate surface coating were used to test the encapsulation capability of the P6c SAPNs. Negatively stained TEM images (Additional file 1: Figure S1a-c) revealed that the thickness of the organic layer around the GNPs was approximately 1 nm manifested by the uranyl acetate staining as a bright ring around the GNPs. The 5 nm GNPs had an average hydrodynamic size of 7.2 nm, while the 10 nm GNPs had an average hydrodynamic size of 14.8 nm (Additional file 1: Figure S1d). The difference between the acclaimed GNP sizes and measured hydrodynamic sizes is attributed to the thickness of the citrate layer and the adsorbed water layer.

**Figure 2:**
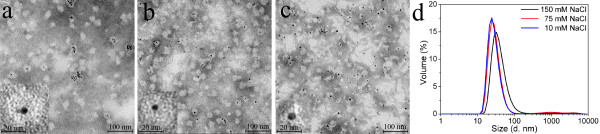
**Encapsulation of 5 nm citrate-coated GNPs into P6c SAPNs.** TEM images of the encapsulation samples at three buffer conditions: (**a**) 20 mM HEPES pH 7.5, 150 mM NaCl, 5% Glycerol; (**b**) 20 mM HEPES pH 7.5, 75 mM NaCl, 5% Glycerol; (**c**) 20 mM HEPES pH 7.5, 10 mM NaCl, 5% Glycerol. (**d**) DLS profiles for volume distribution of hydrodynamic sizes of the encapsulation samples at the three buffer conditions.

The encapsulation results of the 5 nm GNPs are shown in Figure 2. TEM images showed that the 5 nm GNPs with citrate coating were not stable in the refolding buffers of the SAPN, as they contained 75 mM or 150 mM NaCl. The majority of the 5 nm GNPs aggregated before being encapsulated by the P6c SAPNs (Figures 2a and b). TEM images also showed that few 5 nm GNPs were encapsulated by P6c SAPNs, which is demonstrated in the insets of Figures 2a and b. The protein shells around the few encapsulated GNPs were nearly spherical. The thickness of the organic layers around the encapsulated GNPs was approximately 11 nm, which is close to the thickness of the protein shell in the T = 3-like icosahedral model of the P6c SAPNs. This suggests that the encapsulation of the 5 nm GNPs did not disturb the formation of the P6c SAPNs in the refolding buffers containing 75 mM and 150 mM NaCl, although the encapsulation yields were rather low.

In order to avoid aggregation of the 5 nm GNPs, a refolding buffer containing only 10 mM NaCl was used. The 5 nm GNPs were shown to be stable in the refolding buffer containing 10 mM NaCl. However, the protein shells around the encapsulated GNPs became irregular (Figure 2c), which implies that the integrity of the P6c SAPNs was disturbed by the stronger interaction between the protein and the GNPs in buffers with lower ionic strength.

Figure 2d shows the light scattering results of the encapsulation samples in buffers with three different salt concentrations. DLS results show that the average hydrodynamic size of the sample prepared in 150 mM NaCl buffer was larger than those prepared in 75 and 10 mM NaCl buffers. As the samples were mixtures of free GNPs, empty P6c SAPNs, and the GNPs encapsulated by P6c SAPNs, light scattering will yield the average hydrodynamic sizes of all the three kinds of particles. The larger average hydrodynamic size in 150 mM NaCl buffer might be due to the aggregation of the GNPs and/or the larger P6c SAPNs [34].

**Figure 3:**
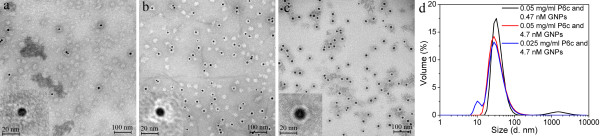
**Encapsulation of 10 nm citrate-coated GNPs into P6c SAPNs.** TEM images of the encapsulation samples that have three different ratios of P6c protein to GNPs, respectively: (**a**) The P6c protein concentration was 0.05 mg/ml (approximately 4 nmol/ml). The 10 nm GNPs concentration was 4.7×10^-4^ nmol/ml (0.47 nM). (**b**) The P6c protein concentration was 0.05 mg/ml. The 10 nm GNPs concentration was 4.7×10^-3^ nmol/ml (4.7 nM). (**c**) The P6c protein concentration was 0.025 mg/ml. The 10 nm GNPs concentration was 4.7×10^-3^ nmol/ml (4.7 nM). The 10 nm GNPs were first diluted in the refolding buffer containing 10 mM HEPES, 75 mM NaCl, 5% glycerol, pH 7.5. Then the denatured P6c protein was refolded in the GNPs solutions using the quick refolding method. After protein refolding, all three samples were dialyzed in a buffer containing 20 mM HEPES, 150 mM NaCl, 5% Glycerol. (**d**) DLS profiles for volume distribution of hydrodynamic sizes of the encapsulation samples.

The encapsulation results of the 10 nm citrate coated GNPs are shown in Figure 3. There were no obvious GNP aggregations observed in the refolding buffer containing 10 mM HEPES pH 7.5, 75 mM NaCl, and 5% glycerol. Therefore, the encapsulation experiment for the 10 nm GNPs was performed in the 75 mM salt buffer using three different ratios of protein to GNPs. After refolding, the samples were dialyzed in a buffer containing 20 mM HEPES pH 7.5, 150 mM NaCl, and 5% glycerol. Figures 3a and b show that there were many empty P6c SAPNs, which implies that there was an excess of P6c protein compared to the concentration of GNPs. Figures 3a and b also show that all GNPs were encapsulated by the P6c SAPNs. The thickness of the protein shells around the encapsulated GNPs was approximately 9 nm, which is close to the theoretical thickness of the protein shell in the T=3-like icosahedral P6c SAPNs. The shapes of the protein shells around GNPs were somewhat irregular, which could be explained by the interactions between the protein chains and the GNP surface.

Figure 3c shows the effect of using lower protein concentration during encapsulation. Although the shapes of the protein shells was more spherical and regular, the thickness of the protein shells around the encapsulated GNPs in Figure 3c was approximately 4.5 nm, which was much thinner than those shown in Figures 3a and b. The difference in the thickness of the protein shells could be due either to insufficient amount of protein for the formation of complete protein shells or to the collapse of the protein on the surface of GNPs.

DLS results (Figure 3d) show that the average hydrodynamic sizes of the encapsulated samples slightly decreased with decreasing ratio of protein to GNPs. When an excess of P6c was used (Figure 3d, black and red line), the average hydrodynamic sizes are actually the average of the empty SAPNs and encapsulated GNPs. TEM images suggested (Figure 3c) that when insufficient amount of protein was used, the protein shells around the encapsulated GNPs became thinner. Therefore the average hydrodynamic size of the encapsulation sample with 0.025 mg/ml P6c and ~4.7×10^-3^ nmol/ml GNPs became smaller. There was a peak around 11 nm (Figure 3d, blue line) present in the encapsulation sample with an excess of GNPs, which is close to the hydrodynamic size of free GNPs. The smaller peak suggests that free GNPs were present in the solution due to an excess of GNPs.

**Figure 4:**
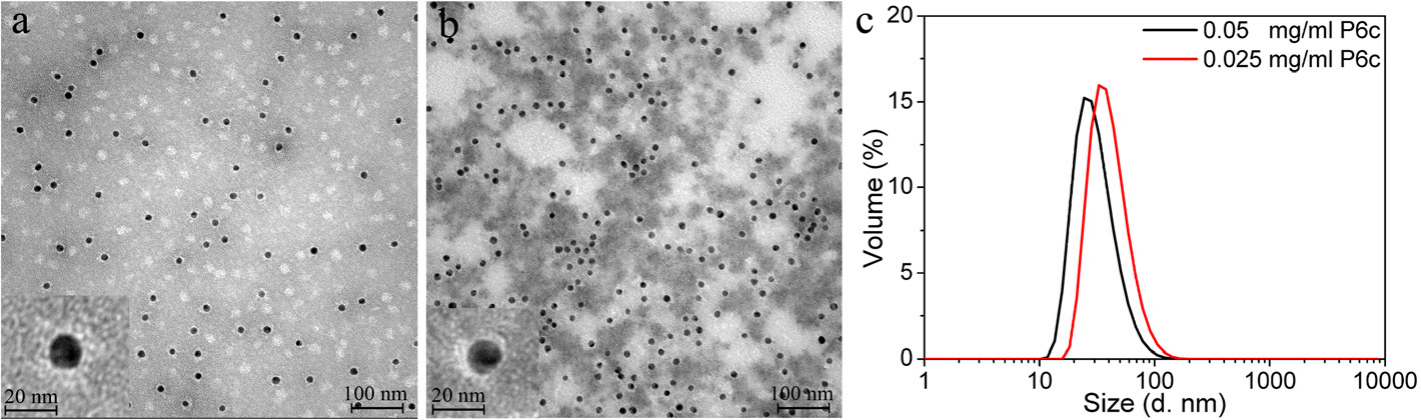
**Encapsulation of 15 nm citrate-coated GNPs into P6c SAPNs.** TEM images of the encapsulation samples that have two different ratios of P6c protein to GNPs, respectively: (**a**) The P6c protein concentration was 0.05 mg/ml (approximately 4 nmol/ml). The 10 nm GNPs concentration was approximately 2.3×10^-3^ nmol/ml. (**b**) The P6c protein concentration was 0.025 mg/ml. The 10 nm GNPs concentration was approximately 2.3×10^-3^ nmol/ml. The denatured P6c protein was refolded in the GNPs solutions using quick refolding method. After protein refolding, all three samples were dialyzed in buffer containing 20 mM HEPES, 150 mM NaCl, 5% Glycerol. (**c**) DLS profiles for volume distribution of hydrodynamic sizes of the encapsulation samples.

The encapsulation results of 15 nm GNPs are shown in Figure 4. Two different ratios of protein to GNPs were used for the encapsulation of 15 nm GNPs with P6c. Figure 4a shows that an excess of P6c proteins was used for encapsulation, as empty P6c SAPNs are visible on the TEM image. The protein shells around the encapsulated GNPs had irregular shapes, and the thickness of the protein shells was approximately 6.5 nm. It is possible that the 15 nm GNPs had strong interactions with the protein chains, which caused the collapse of protein onto the gold surfaces. As shown by Figure 4b, an insufficient amount of P6c was used for encapsulation. The protein shells around the GNPs were incomplete and thinner, compared with the samples shown in Figure 4a. TEM images also showed that the sample with too low protein concentration had a tendency to aggregate (Figure 4b). DLS results (Figure 4c) showed that the average hydrodynamic size of the sample with insufficient amount of protein (0.025 mg/ml P6c) was larger than that with excess protein (0.05 mg/ml P6c). The increased hydrodynamic radius is probably due to the aggregation. This can be observed in TEM images (Figure 4b).

### Encapsulation of PEG-coated GNPs by P6c SAPNs

PEG-coated GNPs of two gold core sizes of 5 and 10 nm were purchased from Nanocs and used for encapsulation. The TEM images of the PEG-coated GNPs with 1% uranyl acetate staining are shown in Additional file 2: Figure S2. The light aureole around the black dots (gold cores) proves the presence of PEG layers coating on the surface of the gold core. Although the shapes of the polymer layers were not regular, the whole particle sizes were considerably larger than the core sizes of the GNPs. Additional file 2: Figure S2c shows the dynamic light scattering profiles of the two PEG-coated GNPs. The hydrodynamic size was 18.6 nm and 21.6 nm for the PEG-coated GNPs with the gold core sizes of 5 nm and 10 nm, respectively. The large hydrodynamic sizes are mainly attributed to the PEG layers and a little to the adsorbed water layer.

**Figure 5:**
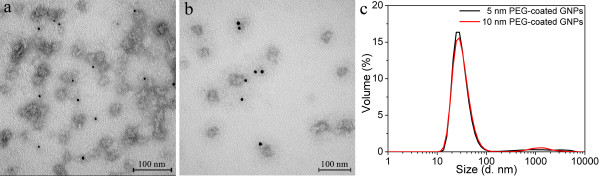
**Encapsulation of PEG-coated GNPs into P6c SAPNs.** TEM images of the encapsulation samples for the PEG-coated gold nanoparticles into P6c SAPNs: (**a**) 5 nm PEG-coated GNPs, (**b**) 10 nm PEG-coated GNPs. The encapsulation of PEG-coated GNPs into P6c SAPNs failed. (**c**) DLS profiles for volume distribution of hydrodynamic sizes of the encapsulation samples. The P6c protein concentration is 0.05 mg/ml for all samples.

The encapsulation results for the two PEG-coated GNPs with P6c are shown in Figure 5. All the GNPs (dark dots) were located outside of the protein nanoparticles, which shows that the PEG-coated GNPs were not encapsulated into the P6c SAPNs. The failure of encapsulation might be due to their large hydrodynamic size. DLS results (Figure 5c) showed similar profiles for both of the encapsulation samples of the 5 nm and 10 nm PEG-coated GNPs. However, light scattering cannot distinguish the PEG-coated GNPs from the P6c SAPNs, since the hydrodynamic size of the PEG-coated GNPs is close to the one of the P6c nanoparticles.

### Encapsulation of polymer-coated GNPs with carboxyl or amine surface functional groups by P6c SAPNs

The P6c SAPNs have a positively charged central cavity due to its arginine residues (R61 in P6c) as shown in a computer model (Figure 1c). Electrostatic interactions between the central cavity and the surface charges from GNPs might exist. Therefore GNPs with different surface charges were tested for encapsulation. The polymer-coated GNPs with carboxyl surface functional groups were purchased from Ocean NanoTech, Inc. The GNPs were coated with amphiphilic polymer bearing carboxyl functional groups. The size of the inorganic core was about 5 nm. The thickness of the organic layers was about 4 nm, as shown in the negatively stained TEM images (Additional file 3: Figure S3a). Dynamic light scattering (Additional file 3: Figure S3c) showed that the hydrodynamic size of the GNPs was 15 nm.

The polymer-coated GNPs with amine surface functional groups were also purchased from Ocean NanoTech, Inc. The GNPs were coated with amphiphilic polymer and PEG coating. Their surface functional group is amine. The size of the inorganic core was about 6 nm. The thickness of the organic layers was about 6 nm (Additional file 3: Figure S3b). Dynamic light scattering (Additional file 3: Figure S3c) showed that the hydrodynamic size of the GNPs was 19.6 nm.

**Figure 6:**
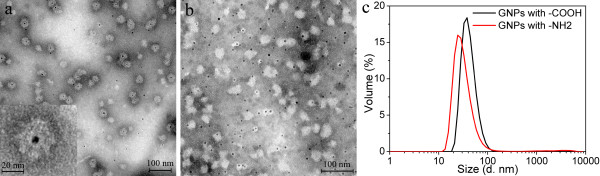
**Encapsulation of polymer-coated GNPs into P6c SAPNs.** TEM images of the encapsulation samples for (**a**) the polymer-coated GNPs with surface carboxylic acid functional groups (GNPs with -COOH), and (**b**) the polymer-coated GNPs with surface amine functional groups (GNPs with –NH2). (**c**) DLS profiles for volume distribution of hydrodynamic sizes of the encapsulation samples. The P6c protein concentration is 0.05 mg/ml for all samples.

The encapsulation results for the two types of GNPs are shown in Figure 6. TEM images (Figure 6a) show that most of the polymer-coated GNPs with carboxyl functional groups were encapsulated into the P6c SAPNs. TEM images also show that there were few empty P6c SAPNs and free GNPs in the sample. The total thickness of the protein layer and the organic layer was approximately 15 nm. Therefore, the thickness of the protein layer was approximately 10 nm. DLS results show the encapsulation sample had an average hydrodynamic size of about 42 nm (Figure 6c, black line). The polymer-coated GNPs with amine functional groups failed to be encapsulated into the P6c SAPNs. TEM images (Figure 6b) show that all the GNPs (dark dots) were located outside of the SAPNs. The different encapsulation results between the two kinds of GNPs could be attributed either to their different surface charges, to their different sizes. But also other factors such as different hydrophobicity could play a role. DLS results show the encapsulation sample had an average hydrodynamic size of about 33 nm (Figure 6c, red line). However, DLS cannot resolve the size difference between the polymer-coated GNPs and the SAPNs.

### Encapsulation of GNPs by P11c SAPNs

The P6c protein has an arginine residue (R61 in P6c, Figure 1a) next to its two-glycine linker, which results in a positively charged central cavity within the P6c SAPN after refolding. When a GNP is encapsulated into the P6c SAPN, the residues around the two-glycine linker are probably in contact with the surface of the GNP (Figure 1e). The environments in the central cavity might affect the encapsulation of GNPs. In order to test the effect of the possible electrostatic interactions between the cavity and the encapsulated GNP, the arginine residue was mutated to a glutamic acid residue (E61 in P11c, Figure 1b). Therefore, P11c SAPNs presumably have an overall negatively charged central cavity.

**Figure 7:**
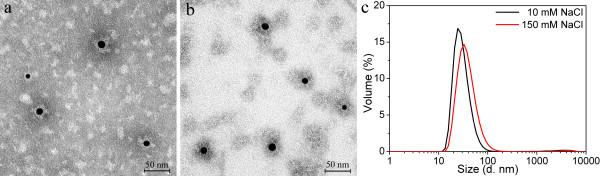
**Encapsulation of 10 nm citrate-coated GNPs into P11c SAPNs.** TEM images of the encapsulation samples at two buffer conditions: (**a**) 20 mM HEPES, 10 mM NaCl, 5% glycerol, pH 7.5; and (**b**) 20 mM HEPES, 150 mM NaCl, 5% glycerol, pH 7.5. The P11c protein concentration is 0.05 mg/ml. (**c**) DLS profiles for volume distribution of hydrodynamic sizes of the encapsulation samples.

The P11c protein was tested for encapsulating the 10 nm citrate-coated GNPs using similar conditions as for the P6c protein. TEM images (Figure 7) show that the 10 nm citrate-coated GNPs were encapsulated by P11c in both buffers containing 10 mM and 150 mM NaCl. The thickness of the protein layer was approximately 8–9 nm for both encapsulation samples. Figure 7c shows the DLS results of the two encapsulation samples. The main factor that contributed to the difference of the DLS results is probably the different sizes of the P11c SAPNs formed in the 10 mM and 150 mM NaCl buffer, since excess amount of empty P11c SAPNs was observed in TEM images (Figure 7).

**Figure 8:**
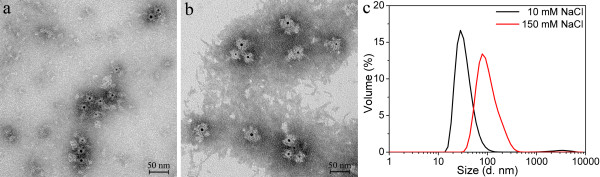
**Encapsulation of polymer-coated GNPs bearing carboxyl functional groups into P11c SAPNs.** TEM images of the encapsulation samples at two buffer conditions: (**a**) 20 mM HEPES, 10 mM NaCl, 5% glycerol, pH 7.5; and (**b**) 20 mM HEPES, 150 mM NaCl, 5% glycerol, pH 7.5. The P11c protein concentration is 0.05 mg/ml. (**c**) DLS profiles for volume distribution of hydrodynamic sizes of the encapsulation samples.

The P11c protein was also used to encapsulate the polymer-coated GNPs with carboxyl functional groups. TEM images (Figure 8) show that the polymer-coated GNPs with carboxyl functional groups were encapsulated into the P11c SAPNs in both buffers. The thickness of the total organic layer was approximately 14–15 nm. Therefore, the thickness of the protein layer was approximately 9–10 nm, which is close to the theoretical thickness of the protein shell in the T=3-like SAPN model. Figure 8c shows the dynamic light scattering results for the two samples. The encapsulation sample in 150 mM NaCl buffer has a large average hydrodynamic size, which can be explained by aggregations in the sample as shown in Figure 8b.

**Figure 9:**
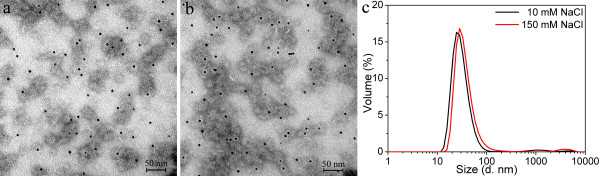
**Encapsulation of polymer-coated GNPs bearing amine functional groups into P11c SAPNs.** TEM images of the encapsulation samples at two buffer conditions: (**a**) 20 mM HEPES, 10 mM NaCl, 5% glycerol, pH 7.5; and (**b**) 20 mM HEPES, 150 mM NaCl, 5% glycerol, pH 7.5. The P11c protein concentration is 0.05 mg/ml. (**c**) DLS profiles for volume distribution of hydrodynamic sizes of the encapsulation samples.

P11c was also used to encapsulate the polymer-coated GNPs with amine functional groups. Figure 9 shows that the GNPs were not located inside the SAPNs. The failure of encapsulation of the polymer-coated GNPs with amine functional groups could be due to their larger sizes.

## Discussion

Our previous work [34] suggested that the majority of the P6c SAPNs are T = 3-like icosahedral particles. Therefore, there might be a size limit for GNPs to be encapsulated. The encapsulation results for the citrate-coated GNPs of three different sizes show that GNPs with hydrodynamic size smaller than about 15 nm (citrate-coated GNPs with 5 nm and 10 nm core sizes) can be encapsulated, although there are aggregation problems with the GNPs in the buffers containing high salt concentrations. The aggregation problem is likely due to increased hydrophobic interactions driven by higher salt concentration. Similarly, the polymer-coated GNPs with carboxyl surface functional groups can be successfully encapsulated into SAPNs, as this type of GNPs also has a hydrodynamic size of 15 nm. On the contrary, the failures in encapsulation of the PEG-coated GNPs (Figure 5) and the polymer-coated GNPs with amine functional groups (Figure 6b) can be attributed to their large hydrodynamic sizes; the three types of GNPs have average hydrodynamic sizes ranging from 18.6 to 21.6 nm.

**Figure 10:**
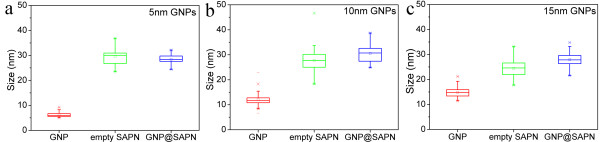
**Comparison of the encapsulation samples from the 5, 10 and 15 nm citrate-coated GNPs.** The sizes of the free gold nanoparticles (GNP, red), empty protein nanoparticles (SAPN, green), and gold nanoparticles encapsulated by protein nanoparticles (GNP@SAPN, blue) measured from the TEM images of the encapsulation samples from (**a**) the 5 nm citrate-coated GNPs (Figure 2a), (**b**) the 10 nm citrate-coated GNPs (Figure 3b), and (**c**) the 15 nm citrate-coated GNPs (Figure 4a), respectively. The sizes were obtained by the program ImageJ, and presented as box-and-whisker plots (the bottom and top of the box are the lower and upper quartiles, the band near the middle of the box is the median, and the ends of whiskers represent the minimum and maximum sizes).

It is ideal for SAPNs to maintain their original icosahedral T = 3-like structure after encapsulation of GNPs. Comparison of the encapsulation samples from the three citrate-coated GNPs indicated that the protein shells coated on larger GNPs are thinner and irregular (Figure 2, 3, 4). The thickness of the protein shell was estimated by comparing the difference between the average size of the SANPs which encapsulate GNPs (GNP@SAPN) and the free GNPs (Figure 10). Although the sizes of GNPs vary, the average sizes of GNP@SAPN change only relatively little (around 30 nm). The average thickness of peptide shells decreased from approximately 11 nm for the 5 nm GNPs, to approximately 9 nm for the 10 nm GNPs, and to only about 6.5 nm for the 15 nm GNPs. The differences in the morphology and thickness of the protein shells imply that the protein chains may to some degree collapse on the surface of the larger GNPs. This collapsing might be due to strong electrostatic interactions between the proteins and the gold surface [36-38]. Based on computer models, the P6c SAPNs have a positively charged central cavity contributed by arginine residues (R61 in P6c, Figure 1). The arginine residues might have electrostatic interactions with citrate on the GNP surface. Therefore, P11c with a glutamic acid residue at position 61 was used to examine the role of the charge of this residue during encapsulation. However, encapsulation results of P11c and P6c with the 10 nm citrate-coated GNPs were well comparable in both buffers with high and with low salt concentrations (10 or 150 mM). This suggests that complex interactions between the protein and citrate-coated GNPs exist and that the protein might to some degree collapse on the surface of GNPs. 

**Figure 11:**
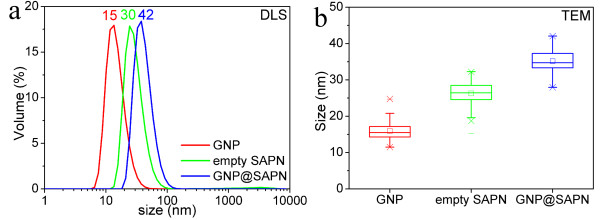
**Analysis of the encapsulation samples from the polymer-coated GNPs.** (**a**) Light scattering profiles of the free polymer-coated gold nanoparticles (GNP, red), empty protein nanoparticles (SAPN, green), and gold nanoparticles encapsulated by protein nanoparticles (GNP@SAPN, blue). (**b**) The sizes of the free GNPs, empty SAPNs and GNP@SAPN from the TEM images of the encapsulation samples were measured by the program ImageJ, presented as box-and-whisker plots.

To alleviate the strong interactions between proteins and gold surfaces, GNPs with PEG or amphiphilic polymer coatings were tested. Compared with the citrate-coated GNPs, the encapsulation samples from the polymer-coated GNPs with surface carboxylic acid functional groups (Figure 6a) possessed more regular protein shells coating the GNPs. The GNPs encapsulating SAPNs became larger than the empty SAPNs (Figure 11). Our previous work [34] shows that the SAPNs can form different species other than the majority of T = 3-like SAPNs. The larger sizes of GNPs encapsulating SAPNs might be due to the formation of larger species, as the hydrodynamic size of the GNPs (~15 nm) is larger than the theoretical size (~10 nm) of the central cavity of T = 3-like SAPNs. The changes in sizes might imply that SAPNs have a certain degree of tolerance for encapsulating GNPs slightly larger than their central cavity. However, there is a limit for the tolerance, as GNPs having average hydrodynamic sizes ranging from 18.6 to 21.6 nm failed to be encapsulated. The thickness of the protein layers coated on the GNPs was estimated as approximately 10 nm (Figure 11), which is close to the theoretical thickness of the protein shells in the model of T = 3-like SAPNs. The morphology of the protein shells suggests that the protein chains didn’t collapse on the surface of the polymer-coated GNPs. Without the collapse of proteins onto the gold surface, the positively charged arginine residues at position 61 might become more important for possible electrostatic interactions, as residues at positions far from the two-glycine linker will not have direct contacts with GNPs. However, the P11c protein again yields similar encapsulation results for the polymer-coated GNPs with surface carboxylic acid functional groups (Figure 8). Therefore, the electrostatic interaction between GNPs and the central cavity may not play a vital role during encapsulation. 

## Conclusions

The success of encapsulation of GNPs into SAPNs allows further functionalization by fusing functional peptides to the nanoparticle-forming protein chains. The SAPNs can present the functional peptides on its surface in an ordered and repetitive ways, when the functional peptides were fused to the termini of the protein chains.

## Methods

### Protein Expression and Purification

The modified pPEP-T vector [39] was kindly provided by the M. E. Müller Institute, Basel, Switzerland. The genes encoding P6c and P11c protein were placed between NcoI and EcoRI restriction sites. The plasmids were then transformed into the Escherichia Coli strain BL21(DE3)pLysS expression cells (Novagen, Madison, WI, USA). The bacteria were incubated at 37°C in Luria Broth (LB) medium in the presence of 200 mg/ml ampicillin and 30 mg/ml chloramphenicol. Expression was induced by adding 1 mM isopropyl β-D-thiogalactopyranoside. After 3 hours of expression, the bacteria were collected by centrifugation at 4000 g for 15 min. The bacterial pellet was resuspended and lysed in a lysis buffer (9 M urea, 100 mM NaH_2_PO_4_, 10 mM Tris, 10 mM β-mercaptoethanol, and pH 8.0) by sonication. The cell debris was removed by centrifugation at 305000 g for 45 min. The supernatant was then incubated with Ni-NTA Agarose beads (Qiagen, Valencia, CA, USA) overnight and then loaded into a column. The protein contaminants were removed by washing the column sequentially with pH buffers 6.3, 5.9 and 5.0, which contain 9 M urea, 100 mM NaH_2_PO_4_, 20 mM sodium citrate, 10 mM imidazole and 10 mM β-mercaptoethanol. The P6c proteins were then eluted by the elution buffer containing 9 M urea, 100 mM NaH_2_PO_4_, 10 mM Tris, 500 mM imidazole, 10 mM β-mercaptoethanol and pH 8.0. The purity of the P6c proteins was verified by sodium dodecyl sulfate polyacrylamide gel electrophoresis.

### Protein refolding procedure

The P6c protein was first denatured in a urea-containing buffer (9 M urea, 20 mM HEPES, 150 mM NaCl, 5% Glycerol, pH 7.5), and then concentrated to 1 mg/ml. The protein was refolded by adding it drop wise to the refolding buffer (20 mM HEPES, 150 mM NaCl, 5% Glycerol, pH 7.5), until the protein concentration reached a concentration of 0.05 mg/ml. The samples were then dialyzed overnight in the refolding buffer to remove the remaining urea.

### GNPs

The citrated-coated gold nanoparticles of the three gold core sizes of 5, 10 and 15 nm, respectively, were purchased from Nanocs Inc., New York, USA. The stock concentrations of the 5, 10 and 15 nm citrate-coated gold nanoparticles were approximately 0.083, 0.0095 and 0.0023 nmole/ml, respectively.

The PEG-coated gold nanoparticles with two gold core sizes of 5 and 10 nm were purchased from Nanocs Inc., New York, USA. The stock concentrations of the 5 and 10 nm PEG-coated gold nanoparticles were approximately 0.083 and 0.0095 nmole/ml, respectively.

The polymer-coated gold nanoparticles with carboxyl or amine surface functional groups were purchased from Ocean NanoTech Inc., AR, USA. Both polymer-coated gold nanoparticle had 5 nm gold cores. The gold nanoparticles with carboxylic acid groups were coated with dodecanethiol and a monolayer of amphiphilic polymer. The zeta potential of these gold nanoparticles is −30 mV to −50 mV (provided by the supplier). The concentration of the gold nanoparticles with carboxylic acid groups was about 5 mg/ml, which gives a concentration of approximately 6.7 nmole/ml. The gold nanoparticles with amine groups were coated with amphiphilic polymer and PEG. The zeta potential of these gold nanoparticles is −10 mV to +10 mV (provided by the supplier). The concentration of the gold nanoparticles with amine groups was about 1 mg/ml, which gives a concentration of approximately 1.3 nmole/ml.

### Encapsulation of citrate-coated gold nanoparticles by P6c SAPNs

The P6c proteins were first denatured in the denaturing buffer (9 M urea, 20 mM HEPES pH 7.5, 150 mM NaCl, 5% glycerol). Then the denatured proteins were concentrated to about 1 mg/ml using the Amicon centrifuge filter (5000 MWCO, Millipore, MA, USA).

The 5 nm citrate-coated nanoparticles were diluted in the refolding buffer to a concentration of approximately 0.0047 nmole/ml. Three different refolding buffers were used for dilution of gold nanoparticles. The refolding buffers were composed of 20 mM HEPES pH 7.5, 5% glycerol, and 10, 75, and 150 mM NaCl, respectively. Then, the denatured P6c protein solution (~1 mg/ml) was added drop wise to the GNP-refolding buffer until the protein concentration reached a concentration of 0.05 mg/ml (3.96 nmole/ml) in the final protein-GNP solution. The protein-GNP solution was then dialyzed overnight against the buffer (20 mM HEPES pH 7.5, 150 mM NaCl, 5% glycerol) to remove the remaining urea.

The encapsulation procedures for the 10 nm citrate-coated gold nanoparticles were similar to that for the 5 nm citrate-coated gold nanoparticles. The 10 nm citrate-coated gold nanoparticles were first diluted in the refolding buffer containing 10 mM HEPES pH 7.5, 75 mM NaCl, and 5% glycerol. Then, the denatured P6c protein solution (~1 mg/ml) was added drop wise to the GNP-refolding buffer until the protein concentration reached a concentration of 0.05 mg/ml (3.96 nmole/ml) in the final protein-GNP solution. The protein-GNP solution was then dialyzed overnight against the buffer (20 mM HEPES pH 7.5, 150 mM NaCl, 5% glycerol) to remove the remaining urea. Three different molar ratios of gold nanoparticles to proteins were used for the encapsulation: (a) The P6c protein concentration was 0.05 mg/ml (approximately 4 nmol/ml). The 10 nm GNPs concentration was 4.7×10^-4^ nmol/ml. (b) The P6c protein concentration was 0.05 mg/ml. The 10 nm GNPs concentration was 4.7×10^-3^ nmol/ml. (c) The P6c protein concentration was 0.025 mg/ml. The 10 nm GNPs concentration was 4.7×10^-3^ nmol/ml.

The encapsulation procedures for the 15 nm citrate-coated gold nanoparticles were also similar to that for the 5 nm citrate-coated gold nanoparticles. The 15 nm citrate-coated gold nanoparticles were first dissolved in the refolding buffer containing 10 mM HEPES pH 7.5, 75 mM NaCl, and 5% glycerol. Then, the denatured P6c protein solution (~1 mg/ml) was added drop wise to the GNP-refolding buffer, until the protein concentration reached a concentration of 0.05 mg/ml (3.96 nmole/ml) in the final protein-GNP solution. The protein-GNP solution was then dialyzed overnight against the buffer (20 mM HEPES pH 7.5, 150 mM NaCl, 5% glycerol) to remove the remaining urea. Two different molar ratios of gold nanoparticles to proteins were used for the encapsulation: (a) The P6c protein concentration was 0.05 mg/ml (approximately 4 nmol/ml). The 10 nm GNPs concentration was approximately 2.3×10^-3^ nmol/ml. (b) The P6c protein concentration was 0.025 mg/ml. The 10 nm GNPs concentration was approximately 2.3×10^-3^ nmol/ml.

### Encapsulation of PEG-coated gold nanoparticles by P6c SAPNs

The PEG-coated gold nanoparticles were first diluted in the refolding buffer containing 10 mM HEPES pH 7.5, 75 mM NaCl, and 5% glycerol to a concentration of approximately 0.0047 nmole/ml. Then, the denatured P6c protein solution (~1 mg/ml) was added drop wise to the GNP-refolding buffer until the protein concentration reached 0.05 mg/ml (3.96 nmole/ml) in the final protein-GNP solution. The protein-GNP solution was then dialyzed overnight against the buffer (20 mM HEPES pH 7.5, 150 mM NaCl, 5% glycerol) to remove the remaining urea.

### Encapsulation of polymer-coated gold nanoparticles with carboxyl or amine surface functional groups by P6c SAPNs

The polymer-coated gold nanoparticles were diluted to approximately 0.01 nmole/ml in the refolding buffer (20 mM HEPES pH 7.5, 150 mM NaCl, 5% glycerol). Then, the denatured P6c protein solution (~1 mg/ml) was added drop wise to the GNP-refolding buffer, until the protein concentration reached a concentration of 0.05 mg/ml (3.96 nmole/ml) in the final protein-GNP solution. The protein-GNP solution was then dialyzed overnight against the buffer (20 mM HEPES pH 7.5, 150 mM NaCl, 5% glycerol) to remove the remaining urea.

### Encapsulation of gold nanoparticles by P11c SAPNs

The encapsulation procedures for gold nanoparticles by the P11c SAPNs were similar to the procedures for the P6c SAPNs. P11c SAPNs were used for the encapsulation of all three kinds of gold nanoparticles mentioned above. The concentration of the P11c proteins was kept as 0.05 mg/ml for all the encapsulation samples. In the final encapsulation samples, approximately 0.0047 nmole/ml of the citrate-coated gold nanoparticles were used. The concentrations of the polymer-coated gold nanoparticles were also approximately 0.01 nmole/ml in their encapsulation samples.

### Dynamic light scattering

The hydrodynamic diameter was determined with a Malvern Zetasizer Nano S equipped with a 633 nm laser. Hellma Quartz cuvettes with a 3 mm light path and centre 9.65 mm were used (Cat. No. 105.251.005-QS). The measurements were performed at 20°C using 80 μl samples. All the samples were filtered once using 0.1 μm Millex-VV filter (Millipore, MA, USA) before measurement. The volume-average hydrodynamic sizes were reported by the Malvern DTS software, version 6.01.

### Transmission Electron Microscopy

A drop of 5 μl sample was placed on a 400 mesh copper grid coated with Formvar/carbon film (Electron Microscopy Sciences, PA, USA) for 1 min. The grid was washed sequentially by three drops of 5 μl distilled water. Then the sample was negatively stained with a drop of 5 μl 1% uranyl acetate (SPI Supplies, PA, USA) for 1 min. Excess stain solution was removed by Whatman filter paper, before the grid was slowly dried at room temperature. Electron micrographs were taken with an FEI Tecnai T12 transmission electron microscope at an accelerating voltage of 80 kV.

The TEM images were first inspected with Photoshop CS4 (Adobe, San Jose, CA). The particles were selected and filled manually using the selection tools in Photoshop CS4, omitting the very small particles or background (area less than 50 nm^2^) and large aggregates (area larger than 5000 nm^2^). Image analysis was then performed with the public domain software ImageJ [40]. Then, the Feret diameter obtained by ImageJ was used to describe the size of the particles.

## Competing interests

PB has an interest in the company Alpha-O Peptides that has patents or patents pending on the technology.

## Authors' contributions

YY performed biophysical experiments developed plasmids to express protein and developed expression and purification methodologies for the nanoparticles and encapsulated nanoparticles with colloidal gold; PB directed the research; YY and PB wrote the manuscript. Both authors read and approved the final manuscript.

## Supplementary Material

Additional file 1 **Figure S1.** Citrate-coated GNPs. TEM images of the citrate-coated GNPs with 1% uranyl acetate staining: (**a**) 5 nm GNPs, (**b**) 10 nm GNPs, and (**c**) 15 nm GNPs. The thickness of the organic layer is approximately 1 nm. (**d**) DLS profiles of the 5 nm and 10 nm GNPs with citrate coating. Click here for file

Additional file 2 **Figure S2.** PEG-coated GNPs. TEM images of the PEG-coated GNPs with 1% uranyl acetate staining: (**a**) PEG-coated GNPs with core size of 5 nm, (**b**) PEG-coated GNPs with core size of 10 nm. PEG-coated GNPs are too large to be encapsulated. (**c**) DLS profiles of the PEG-coated GNPs with core size of 5 nm and 10 nm respectively. Click here for file

Additional file 3 **Figure S3.** Polymer-coated GNPs. TEM images of the polymer-coated GNPs with 1% uranyl acetate staining: of (a) the gold nanoparticles with surface carboxylic acid functional groups, and (b) the gold nanoparticles with surface amine functional groups. (c) DLS profiles of the polymer-coated GNPs with surface carboxylic acid functional groups, and the polymer-coated GNPs with surface amine functional groups, respectively. Click here for file
