# Supplementary figures and images for: Encapsulation of gold nanoparticles into self-assembling protein nanoparticles
Source: J Nanobiotechnology. 2012 Oct 31;10:42. doi: 10.1186/1477-3155-10-42 (PMC3502577; doi:10.1186/1477-3155-10-42)

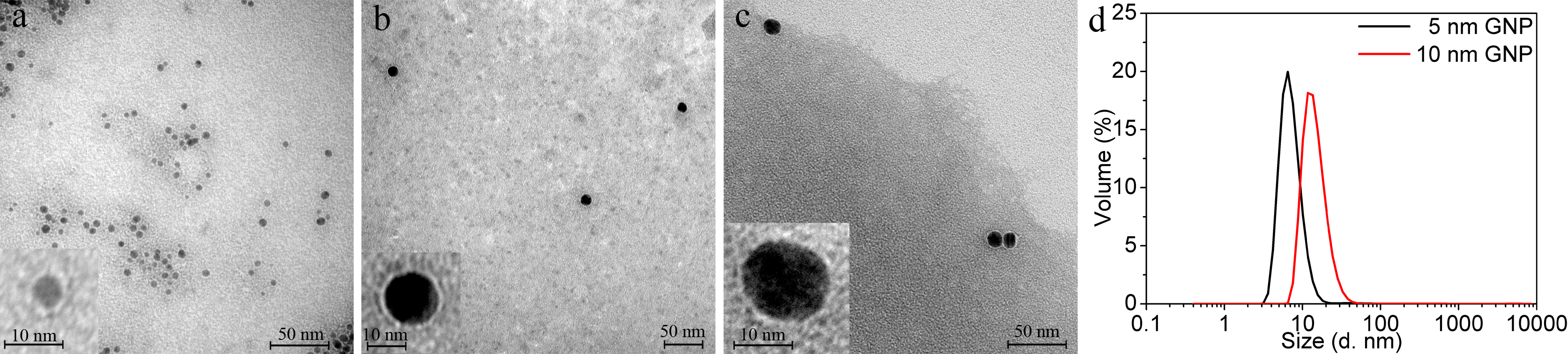

Supplement: Additional file 1 — Figure S1. Citrate-coated GNPs. TEM images of the citrate-coated GNPs with 1% uranyl acetate staining: (a) 5 nm GNPs, (b) 10 nm GNPs, and (c) 15 nm GNPs. The thickness of the organic layer is approximately 1 nm. (d) DLS profiles of the 5 nm and 10 nm GNPs with citrate coating. [file 1477-3155-10-42-S1.tiff]

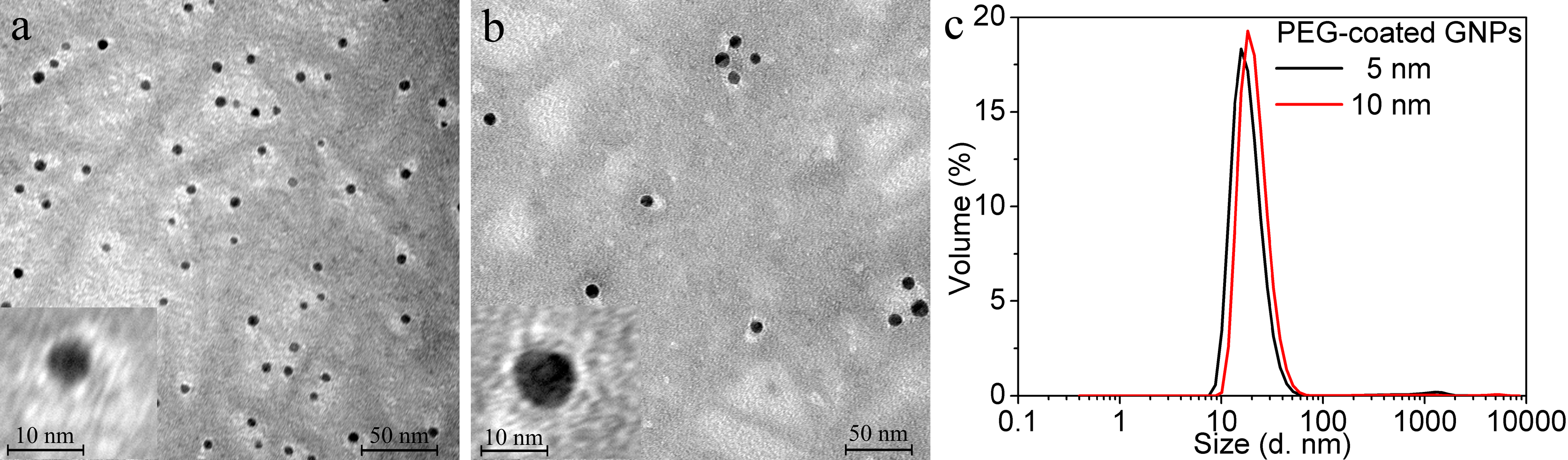

Supplement: Additional file 2 — Figure S2. PEG-coated GNPs. TEM images of the PEG-coated GNPs with 1% uranyl acetate staining: (a) PEG-coated GNPs with core size of 5 nm, (b) PEG-coated GNPs with core size of 10 nm. PEG-coated GNPs are too large to be encapsulated. (c) DLS profiles of the PEG-coated GNPs with core size of 5 nm and 10 nm respectively. [file 1477-3155-10-42-S2.tiff]

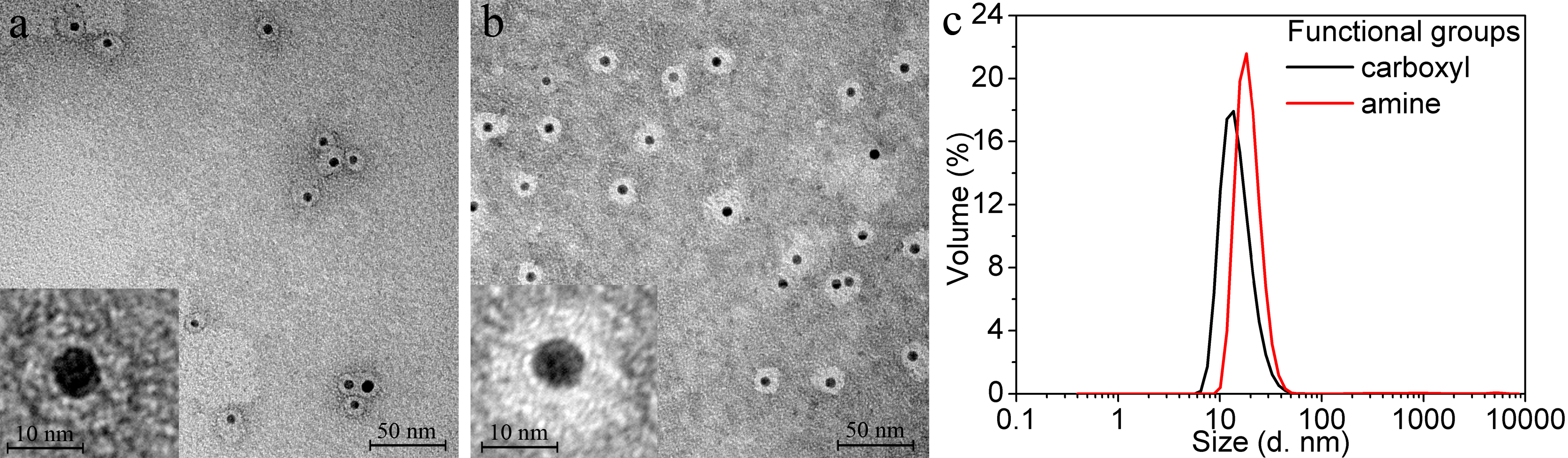

Supplement: Additional file 3 — Figure S3. Polymer-coated GNPs. TEM images of the polymer-coated GNPs with 1% uranyl acetate staining: of (a) the gold nanoparticles with surface carboxylic acid functional groups, and (b) the gold nanoparticles with surface amine functional groups. (c) DLS profiles of the polymer-coated GNPs with surface carboxylic acid functional groups, and the polymer-coated GNPs with surface amine functional groups, respectively. [file 1477-3155-10-42-S3.tiff]
